# Functional assessment of protein variants in structured domains by fluorescence cross-correlation spectroscopy

**DOI:** 10.1038/s41598-025-34563-6

**Published:** 2026-01-02

**Authors:** Àngels Mateu-Regué, Luca Mariani, Frederik Otzen Bagger, Muthiah Bose, Finn Cilius Nielsen

**Affiliations:** 1 Center for Genomic Medicine, Copenhagen University Hospital, Rigshospitalet Blegdamsvej 9, Copenhagen, 2100 Denmark; 2 Department of Clinical Medicine, University of Copenhagen, Blegdamsvej 3, Copenhagen, 2200 Denmark

**Keywords:** Fluorescence Cross-Correlation spectroscopy, *In vivo* protein complex formation, BRCA1, Variants of uncertain significance, Variant classification, Monogenic diseases, Cancer, Microscopy

## Abstract

With the expanding catalogue of novel disease-genes, there is an increasing need to establish the significance of potential disease-causing variants. Based on the idea that pathogenic variants in structured protein domains disturb folding and association with macromolecular assemblies, we employed Fluorescence Correlation and Cross-Correlation Spectroscopy (FCS and FCCS) to assess in vivo protein complex formation. Since the molecular underpinning of BRCA-associated breast and ovarian cancers is well defined and data from a recent genome editing screening allowed us to compare variant binding data with a reliable functional HRD test in addition to ClinVar and AlphaMissense data, we examined the binding of mutated full-length BRCA1 or isolated RING and BRCT domains to BARD1 and RBBP8, respectively. The results demonstrate that FCCS, whether applied to full-length BRCA1 in live cells and/or to isolated domains in cellular lysates, identified pathogenic *BRCA1* RING or BRCT domain variants. We moreover demonstrate the feasibility of employing FCCS for analysis of HNPCC-related factor MSH2 and MEN1 factor Menin variants in combination with DNA mismatch repair factor MSH6 and transcription factor JUND, respectively. We propose that FCCS may be an appealing complement to current clinical procedures for classifying variants for many monogenic diseases, given its generic nature and ease of use.

**Supplementary Information:**

The online version contains supplementary material available at 10.1038/s41598-025-34563-6.

## Introduction

The rapid advancements in genomics have enhanced our comprehension of the genetic underpinnings of various rare diseases. With an ever-expanding catalogue of novel disease-associated genetic variations, there is a pressing need to establish the clinical significance of potential disease-causing variants. Traditionally, variant classification depended on the nature of the variant and its known association or co-segregation with a specific disease. However, co-segregation is not always feasible, leading to the contemporary utilization of ACMG/AMP guidelines^1^, which based on data from functional studies, segregation analyses, or clinical correlations categorize variants as pathogenic, likely pathogenic, variants of uncertain significance (VUSs), likely benign, or benign. VUSs, in particular, pose a significant clinical challenge as they leave patients in a state of uncertainty. To address this challenge, the development of large-scale variant databases, enhanced computational predictive tools like AlphaMissense^2^, and the advancement of functional analyses have represented significant steps forward. Two notable examples of functional analyses involve the early use of minigenes^3^ to investigate mutations affecting mRNA splicing, and more recently, systematic screening approaches such as the BRCA1 saturation genome editing screening (SGE)^4^, aimed at assessing homologous recombination deficiency. However, there is currently no single, universally applicable approach for functional testing of protein variants, which impedes the application of functional analyses in the clinical environment.

Proteins are fundamental to cellular processes, and it is estimated that the majority of proteins within a given proteome exert their function as part of complexes with other factors^5^. Assembly is governed by conserved structured domains within the proteins and pathogenic variants frequently disrupt the structure of these domains, leading to misfolding of the proteins and destabilization of the functional units^6–9^. Various methods, such as affinity purification coupled with mass spectrometry (AP-MS) and yeast two-hybrid (Y2H) assays, have effectively been employed to characterise protein-protein complexes. While these methods have contributed significantly to our understanding of cellular protein networks, they are labour-intensive and may not always be readily adaptable for clinical analyses.

**Fig. 1:**
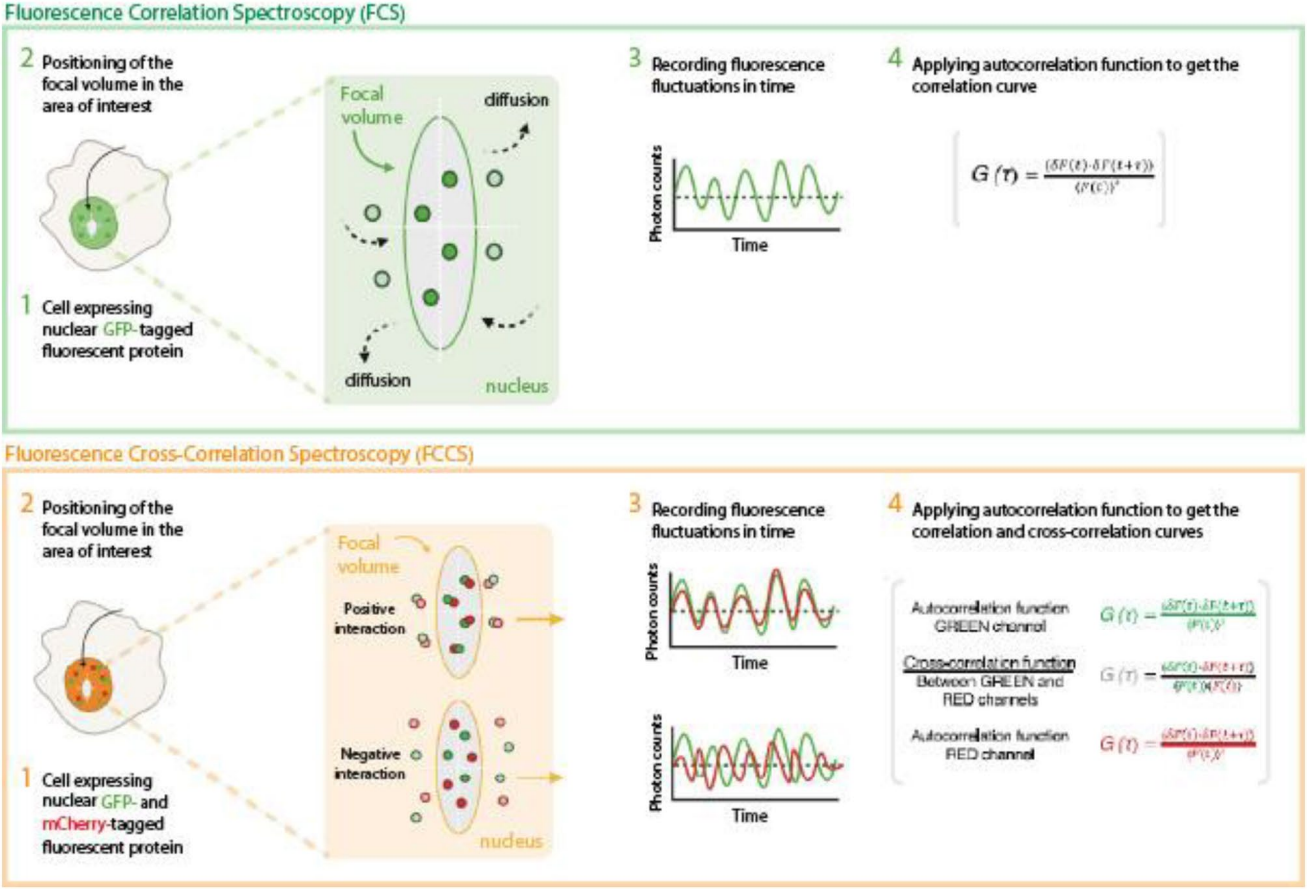
Schematic representation of Fluorescence Correlation Spectroscopy (FCS) and Fluorescence Cross-Correlation Spectroscopy (FCCS) methods used in the study. Upper panel - FCS measurements are performed either in cells expressing a protein of interest fused to a GFP tag or in cell lysates from cells expressing the GFP fusion protein. Focal volume is positioned in a specific cellular location (live cell FCS) or in a fluorescent protein solution (cell lysate FCS). Fluorescence fluctuations are recorded and analysed by the autocorrelation function, which generates an autocorrelation curve, that is used to determine the diffusion time of the examined GFP-tagged protein. Lower panel - FCCS is based on the combined FCS measurements of two spectrally-distinct fluorescent proteins, in this case GFP and mCherry. The autocorrelation function is used for analysing diffusion of GFP- and mCherry-tagged proteins and also applied between channels, generating the cross-correlation curve. Interacting GFP- and mCherry-tagged proteins diffuse synchronously through the focal volume, so the cross-correlation curve is positive. In contrast, non-interacting GFP- and mCherry-tagged proteins diffuse independently from each other yielding a flat cross-correlation curve. The maximum possible cross-correlation occurs when a molecule labeled with one fluorophore interacts with a molecule labelled with the other fluorophore. This interaction cannot happen more frequently than the presence of the most abundant molecule. Therefore, the FCCS cross-correlation curve can only reach a maximum value corresponding to the concentration of the most abundant molecule.

Fluorescence Correlation and Cross-Correlation Spectroscopy (FCS and FCCS) offer alternative generic approaches to rapidly and precisely assess protein diffusion, stoichiometry, and complex formation (Fig. 1)^10,11^. FCS is based on the recording of fluorescence fluctuations produced by labelled molecules or proteins entering and exiting a small focal volume and analysing these fluctuations through autocorrelation. FCCS, on the other hand, leverages cross-correlation between molecules or proteins labelled with distinct spectral markers to determine if two factors associate or are part of the same macromolecular complex^10^. FCS and FCCS are versatile techniques that have been widely applied to investigate a broad range of cellular processes, including protein–protein and protein–nucleic acid interactions, receptor–ligand binding, molecular diffusion dynamics, chromatin organization, transcription factor dynamics, protein complex formation, and aggregation, demonstrating their utility across diverse biological contexts^12–17^. They offer single-molecule sensitivity and can analyse proteins and their interactions both in live cells and in vitro. Although specialized microscopy instrumentation and trained personnel are required, individual measurements can be acquired within minutes, which makes these methods particularly valuable in both clinical and research settings, as they combine speed with quantitative precision and reproducibility.

Based on the idea that pathogenic variants in structured protein domains disrupt macromolecular assemblies, we employed FCCS to rapidly assess in vivo protein complex formation and thereby functionally classify variants. We employed hereditary breast and ovarian cancer (HBOC) as a model disease because the molecular underpinning is well defined^18^. Moreover, data from massively parallel assays allow us to compare our results with other functional tests^4,19^. HBOC susceptibility genes are involved in homologous recombination repair (HRR), replication fork stability, DNA replication, and cell cycle checkpoint pathways. HRR is governed by a large assembly of factors, including BRCA1 and BRCA2 (*BR*east *CA*ncer genes *1* and *2*), as well as PALB2 (*PA*rtner and *L*ocalizer of *B*RCA*2*), ATM (*A*taxia *T*elangiectasia *M*utated), CHEK2 (*CHE*eckpoint *K*inase *2*), RAD51C (*RAD51* Paralog *C*), RAD51D (*RAD51* Paralog *D*), BARD1 (*B*RCA1 *A*ssociated *R*ING *D*omain *1*), RBBP8 (*R*etino*B*lastoma-*B*inding *P*rotein *8*) and BRIP1 (*BR*CA1-*I*nteracting *P*rotein *1*). BRCA1 serves as scaffold for a number of these factors and pathogenic mutations are frequently found in the BRCA1 RING and BRCT domains, that among other factors associate with BARD1 and RBBP8, respectively^20–22^. We show that FCCS, whether applied to full-length BRCA1 in live cells and/or to isolated domains in cellular lysates, reliably identify known *BRCA1* RING or BRCT pathogenic variants. We also demonstrate the feasibility of employing the method for the analysis of hereditary non-polyposis colorectal cancer (HNPCC)-related factor MSH2 (*M*ut*S H*omolog *2*)^23^ and MEN1 factor Menin^24^ in combination with DNA mismatch repair factor MSH6 (*M*ut*S H*omolog *6*)^25^ and transcription factor JUND (*JunD* Proto-Oncogene)^26^, respectively. Since the analysis can be completed in just a few hours, FCCS could complement current clinical procedures in classifying genetic variants. Given its generic nature and design, we envision that FCCS could serve as a valuable tool for variant classification in a wide variety of monogenic diseases.

## Materials and methods

### Cell lines

HeLa cells (ATCC^®^ CCL-2™) were cultured in Dulbecco’s Modified Eagle Medium (DMEM), high glucose, no glutamine, no phenol red (Thermo Fisher Scientific, Cat. No. 31053028), supplemented with 1X GlutaMAX™ (Thermo Fisher Scientific, Cat. No. 35050038), 1 mM sodium pyruvate (Thermo Fisher Scientific, Cat. No. 11360039), 10% FBS (Biowest, Cat. No. BWSTS1810) and 1% penicillin/streptomycin (Thermo Fisher Scientific, Cat. No. 15070063) and were grown in a humidified incubator at 37 °C and 5% CO_2_.

### Plasmids

The coding sequence of *BRCA1* was PCR-amplified and cloned into pEGFP-C1 (Clontech) by restriction site digestion (SalI/SacII) and ligation. *BARD1* and *RBBP8* coding sequences were also PCR-amplified and cloned into pmCherry-C1 (Clontech). The sequences encoding for amino acids 1–109 and 1650–1864 of BRCA1 (as recommended by ClinGen *BRCA1* Variant Curation Expert Panel) were separately cloned into pEGFP-C1 to generate vectors expressing the EGFP-tagged RING and BRCT trimmed domains, respectively. EGFP was PCR-amplified from pEGFP-C1 and cloned into pmCherry-C1 by restriction enzyme digestion in order to obtain the pmCherry-EGFP fusion protein^27^. All inserts were confirmed by Sanger sequencing. Scale-up of plasmid DNA was performed with GeneJET Plasmid Midiprep Kit (Thermo Fisher Scientific, Cat. No. K0481).

### Cell seeding and plasmid transfection

For experiments in live cells, 200,000 HeLa cells were seeded in 4-well chambered glass-bottom coverslips (µ-Slide 4 Well Glass Bottom, Ibidi, Cat. No. 80427). After 4–5 h, cells were transfected with relevant plasmids using FuGene HD transfection reagent (Promega, Cat. No. E2311). Briefly, 3 µl of FuGENE^®^ HD Transfection reagent were added to 50 µl of Opti-MEM^TM^ (Thermo Fisher Scientific, Cat. No. 31985062). Next, 500 ng of plasmid DNA were added, and the mixture was incubated for 15 min before addition to the wells. To achieve similar protein expression levels, GFP and mCherry vectors were mixed at a 2:1 ratio. For experiments in lysates, 250,000 cells were seeded per well in 6-well plastic-bottom plates (Nunc™ Cell-Culture Treated Multidishes, Thermo Fisher Scientific, Cat. No. 140675). After 4–5 h, cells were transfected with relevant plasmids using FuGene^®^ HD transfection reagent. Briefly, 18 µl of FuGENE^®^ HD Transfection reagent were added to 600 µl of Opti-MEM^™^. Next, 1 µg of plasmid DNA was added and the mixture was incubated for 15 min before 100 µl were added to each well. To achieve similar protein expression levels, GFP and mCherry vectors were mixed either at a 1:2 ratio (RING domain: BARD1), 1:4 ratio (BRCT domain: RBBP8), 1:1 ratio (MSH6:MSH2), or 1:1 ratio (Menin: JUND).

### Confocal microscopy imaging

HeLa cells were seeded in 35 mm glass bottom dishes (No. 1.5 Coverslip 14 mm G, uncoated, MatTek Corporation, Cat. No. P35G-1.5–14.5-C), transfected with GFP or/and mCherry vectors and subsequently imaged ∼24 h after transfection. Confocal images were obtained using a Zeiss LSM780 confocal microscope with a Plan-Apochromat 63x/1.4 NA oil objective.

### Fluorescence correlation spectroscopy (FCS)

FCS measurements were recorded the day after transfection, selecting cells where the expression levels of GFP and mCherry-tagged proteins were low. FCS measurements were performed with a Zeiss LSM780 confocal microscope using a C-Apochromat 40×/1.2 W Corr M27 objective and Immersion oil Immersol W 2010 (Zeiss). GFP and mCherry measurements were performed with an Argon laser with a 488 nm excitation wavelength (0.1% laser power) and a DPSS laser with a 561 nm excitation wavelength (0.1% laser power), respectively. GFP fluorescence was captured with a detection window of 482–553 nm and mCherry fluorescence was captured with a detection window of 590–695 nm, as described earlier^27^. Before each measurement, average molecular count rate (kHz per molecule) was assessed at different laser powers to ensure that fluorescence count signal was linear with laser power and not in saturation. Measurements were performed at randomly selected volumes in the cell nucleus, excluding nucleoli and other dense structures, for 1.5 min in 30 s intervals. The coverslip was taken from the incubator (37 °C) and sealed with parafilm, before it was mounted on the microscope. The microscope measurements were taken at room temperature and coverslips were kept in the incubator until analyses. The experimental autocorrelation curves were obtained and analysed in ZEN 2011 software (Zeiss). The fits of the different models to the experimental data were also performed in ZEN 2011, using their in-built models as described earlier^27^.

### Fluorescence cross-correlation spectroscopy (FCCS)

FCCS measurements in live cells were taken the day after transfection, selecting cells where the expression levels of GFP and mCherry-tagged proteins were low. Recordings were performed in a Zeiss LSM780 confocal microscope using a C-Apochromat 40×/1.2 W Corr M27 objective and Immersion oil Immersol W 2010 (Zeiss). GFP and mCherry measurements were performed with an Argon laser with a 488 nm excitation wavelength (0.1% laser power) and a DPSS laser with a 561 nm excitation wavelength (0.1% laser power), respectively. GFP fluorescence was captured with a detection window of 482–553 nm and mCherry fluorescence was captured with a detection window of 590–695 nm. Before each measurement, average molecular count rate (kHz per molecule) was assessed at different laser powers to ensure that fluorescence count signal was linear with laser power and not in saturation. Measurements were performed at randomly selected volumes in the cell nucleus for 1.5 min in 30 s intervals. As mentioned above nucleoli and other dense structures were excluded. The coverslip was taken from the incubator (37 °C) and sealed with parafilm, and measurements of two wells (2/4) were taken 5–30 min later. Coverslip was placed back to the incubator for 30 min before taking the FCCS measurements of the remaining 2 wells. FCCS was performed on 5–10 cells for each tested variant, as indicated in Supplementary Fig. 2. Experimental autocorrelation and cross-correlation curves were obtained and analysed in ZEN 2011 software (Zeiss). Cross-correlation and correlation amplitude values, needed to calculate the cross-correlation/correlation ratios, were extracted from the average of the amplitude values G(τ) in the interval from time points 8.00E-06–4.00E-04 from either curve in the area of maximum amplitude. The cross-correlation (CC)/autocorrelation (AC) ratio was calculated with the following formula: CC/AC ratio = [G(τ)CC – 1]/[G(τ)AC – 1].

FCCS on lysates was also performed the day after transfection. Cells were lysed at room temperature in 100 µl of lysis buffer containing 20 mM Tris-HCl pH 7.5, 140 mM KCl, 1.5 mM MgCl_2_, 1mM DTT and 0.5% NP-40 supplemented with 1:300 mammalian protease inhibitor cocktail (Sigma). Cell lysates were briefly centrifuged at 800 x g for 1 min and supernatant was transferred to a 35 mm glass bottom dish (No. 1.5 Coverslip 14 mm G, uncoated, MatTek Corporation, Cat. No. P35G-1.5–14.5-C) before being subjected to FCCS. All the settings described above for FCCS analyses in live cells were also used for FCCS in lysates, except for the laser power, which was optimized as follows. For GFP-tagged RING domain variants and mCherry-tagged BARD1: GFP and mCherry measurements were performed with an Argon laser with a 488 nm excitation wavelength (0.3% laser power) and a DPSS laser with a 561 nm excitation wavelength (0.5% laser power), respectively. For GFP-tagged BRCT domain variants and mCherry-tagged RBBP8: GFP and mCherry measurements were performed with an Argon laser with a 488 nm excitation wavelength (0.4% laser power) and a DPSS laser with a 561 nm excitation wavelength (0.4% laser power), respectively. The same laser settings were used for GFP-tagged MSH2 and mCherry-tagged MSH6 and GFP-tagged Menin and mCherry-tagged JUND.

## Results

### Fluorescence correlation spectroscopy of nuclear GFP-BRCA1

**Fig. 2:**
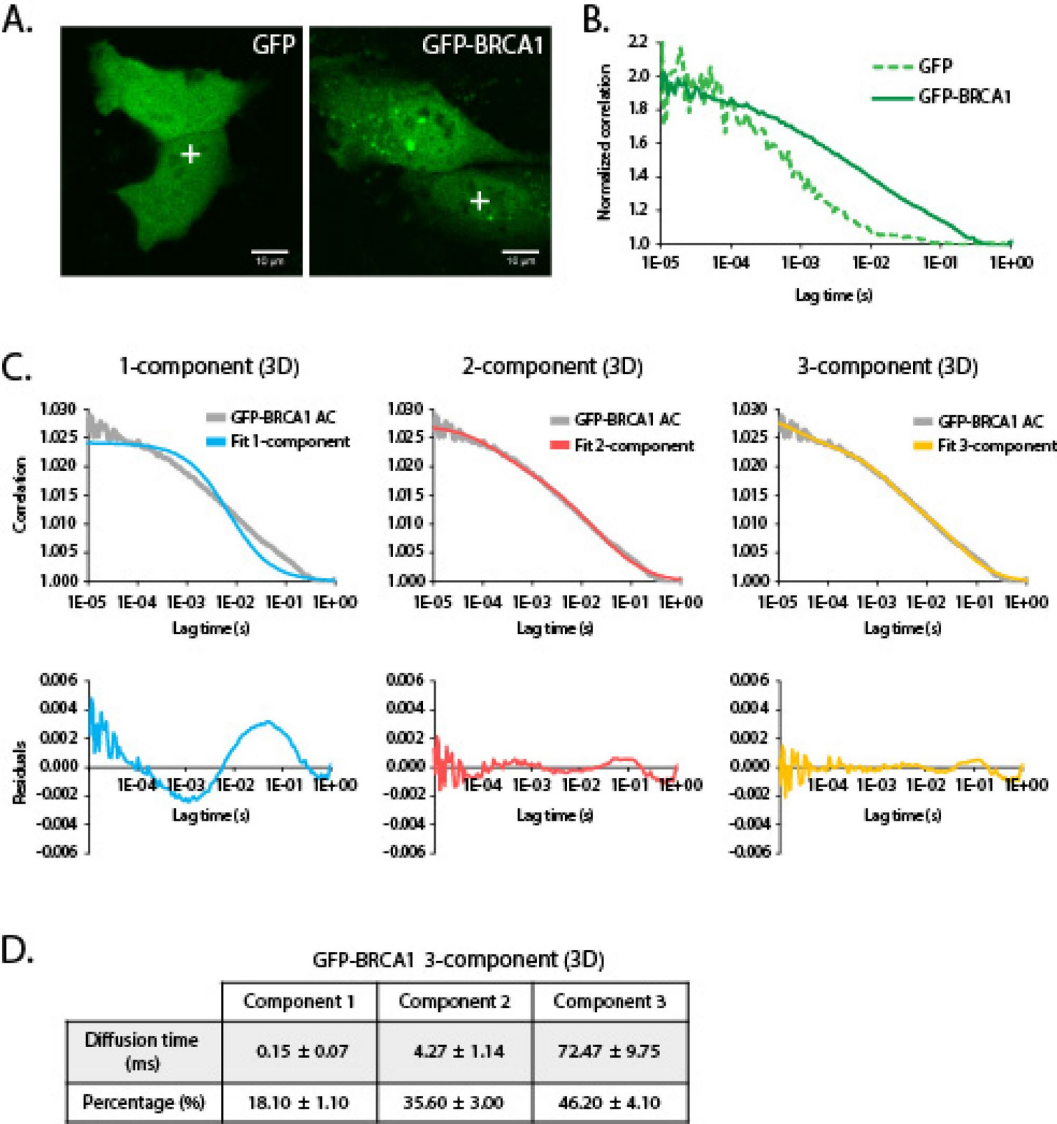
Complex formation of GFP-BRCA1 in live cells. (**A**) Confocal images of HeLa cells expressing GFP and GFP-tagged BRCA1. GFP exhibits a diffused fluorescent pattern throughout the cell while GFP-BRCA1 shows a prominent nuclear localisation of the tagged protein. Crosses in the nuclear space show how focal volumes were arbitrarily positioned in the nucleoplasm to record the fluorescent measurements. (**B**) Normalized autocorrelation curves for GFP and GFP-BRCA1 reveal a slower average diffusion rate for GFP-BRCA1 compared to free GFP. (**C**) Fitting of the GFP-BRCA1 autocorrelation curve (AC) to free (3D) 1-, 2- and 3-component diffusion models (top) and their corresponding residuals (bottom). (**D**) Diffusion time and percentage (%) of GFP-BRCA1 fitting to a free (3D) 3-component diffusion model.

In order to assess whether FCS was able to characterise the nuclear diffusion of BRCA1, HeLa cells were transiently transfected with a plasmid encoding either GFP-BRCA1 or GFP alone. Since autocorrelation is inversely correlated with the concentration of the fluorescent molecule, measurements were obtained from cells exhibiting low expression of these factors (Fig. 2A). As illustrated by the normalized autocorrelation curves, nuclear GFP-BRCA1 diffuses approximately 8 times slower than GFP (Fig. 2B), indicating that GFP-BRCA1 is likely to be part of a larger assembly. In agreement with this, a subsequent fitting of the autocorrelation curve to models of 1, 2 or 3 components showed that the GFP-BRCA1 autocorrelation curve did not fit a free (3D) 1-component diffusion model (blue line) (Fig. 2C, D). The poor fitting is illustrated by the residuals, which show the difference between the experimental data and the model fit. A better fit was observed after employing a 2-component diffusion model (red line), although the fluctuation of the residuals was not completely random and fluctuating around zero. In contrast, fitting to a 3-component diffusion model (yellow line) generated an almost random distribution of the residuals, indicating that nuclear GFP-BRCA1 associates with a number of nuclear proteins.

### Fluorescence cross-correlation spectroscopy of BRCA1 binding to BARD1 and RBBP8

**Fig. 3:**
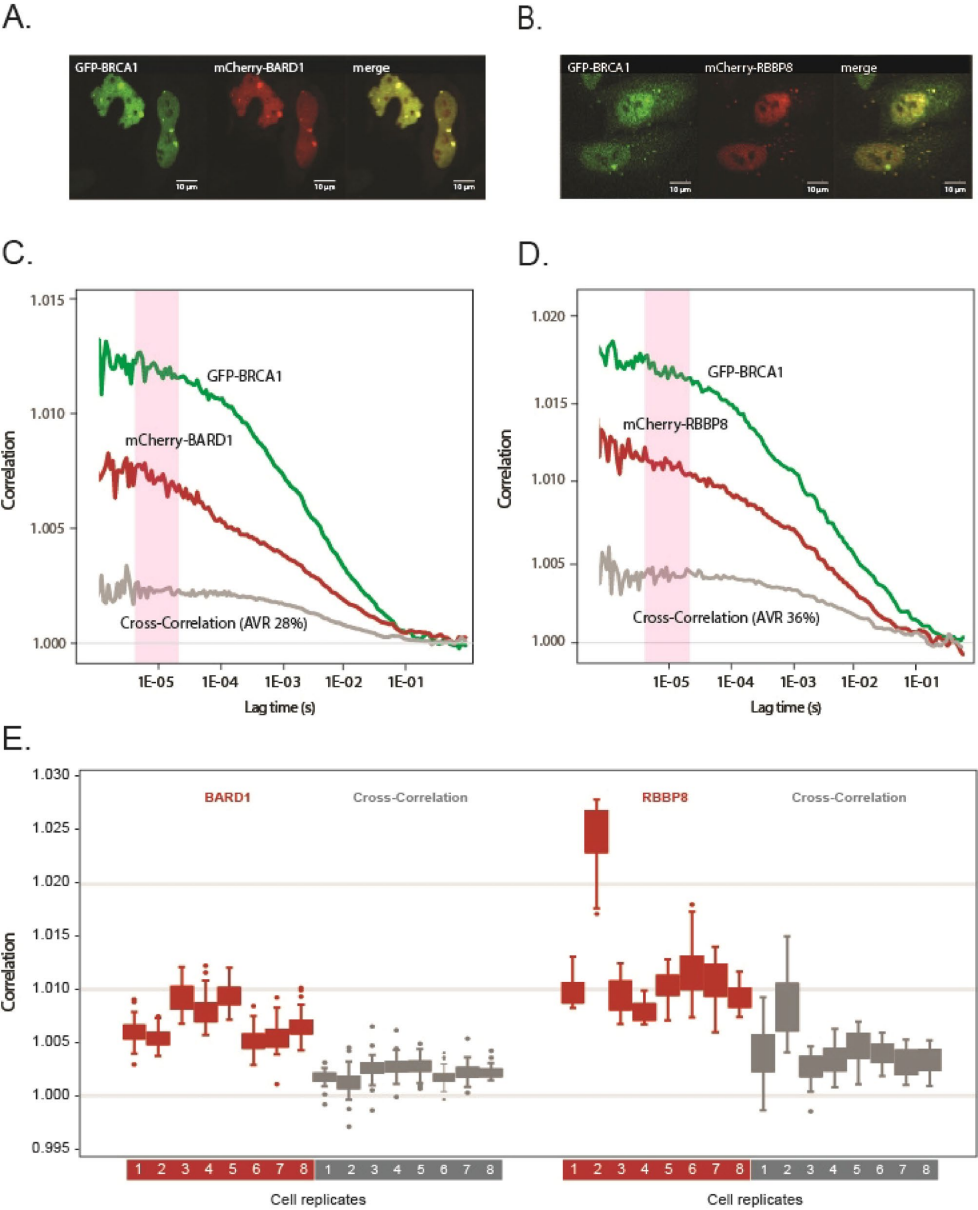
Association of BRCA1 with BARD1 and RBBP8/CtIP in live cells. (**A**) Confocal images of HeLa cells expressing GFP-BRCA1 and mCherry-BARD1. (**B**) Confocal images of HeLa cells expressing GFP-BRCA1 and mCherry-RBBP8/CtIP. (**C**) Autocorrelation and cross-correlation curves obtained from GFP-BRCA1 and mCherry-BARD1 nuclear measurements. (**D**) Autocorrelation and cross-correlation curves obtained from GFP-BRCA1 and mCherry-RBBP8 nuclear measurements. The pink columns in (**C**) and (**D**) show the range of autocorrelation values that were averaged to determine the plateau of the curves. (**E**) The distribution and variation of absolute averaged autocorrelation values for BARD1 and RBBP8 plateaus, and their corresponding cross-correlation curves, are presented across eight distinct cells (labeled 1–8).

BRCA1 serves as a critical scaffold for the assembly of complexes involved in HRR. Its N-terminal RING domain interacts with BARD1, while the C-terminal BRCT domain, along with other factors, associates with RBBP8 (CtIP)^20–22^. We therefore exploited the possibility to detect and quantify complex formation among these factors using Fluorescence Cross-Correlation Spectroscopy. Cells were co-transfected with plasmids encoding GFP-BRCA1 and mCherry-BARD1 or mCherry-RBBP8, respectively. Nuclear FCCS measurements were performed in the nucleoplasm avoiding the nucleolus, matrix and dense foci with conceivably immobile GFP-BRCA1. For each measurement, the GFP-BRCA1 and mCherry-BARD1 or mCherry-RBBP8 autocorrelation curves and the cross-correlation curve were obtained. Measurements were routinely obtained from 5 to 10 different cells and in each cell, we performed at least 3 measurements of 30 s. Unstable correlation curves distorted by bleaching or instability of the stage were omitted from the analysis. We also favoured cells exhibiting low levels of the expressed factors in order to reach correlations above 1.005, although this was not always feasible. GFP-BRCA1, mCherry-BARD1 and mCherry-RBBP8 are mainly nuclear, although a significant fraction of BRCA1 and BARD1 also remained in the cytoplasm. In cells exhibiting nuclear speckles the factors co-existed, indicating they were part of the same complexes (Fig. 3A, B). The graphs in Fig. 3C, D show the averaged autocorrelation curves for GFP-BRCA1, mCherry-BARD1 or mCherry-RBBP8 and the corresponding cross-correlation curve, respectively. The averaged cross-correlation values of mCherry-BARD1 and -RBBP8 with GFP-BRCA1 were 28% (STDEV 8%) and 36% (STDEV 11%), respectively. For comparison, a mCherry-GFP fusion protein^27^ exhibited a cross-correlation of 33% (STDEV 5%) (Supplementary Fig. 1). Figure 3E shows the variation of the correlation amplitudes among different cells. At the described inter-replicate STDEV, the assay may detect a reduced cross-correlation of > 35% (*P* ≤ 0.05). Since the correlation readings obtained in live cells are relatively low and variable compared to measurements in solutions (see below), we averaged correlation values over a series of 20 readings (time points 8.00E-06–4.00E-04 s) in order to establish the plateau of the correlation curves, as indicated in Fig. 3C, D. Only replicates exhibiting a STDEV of < 0.01 among the plateau values were used for the analysis, thus readings showing high variation at the plateau were excluded.

### FCCS analyses of BRCA1 RING and BRCT domain variants in live cells

**Fig. 4:**
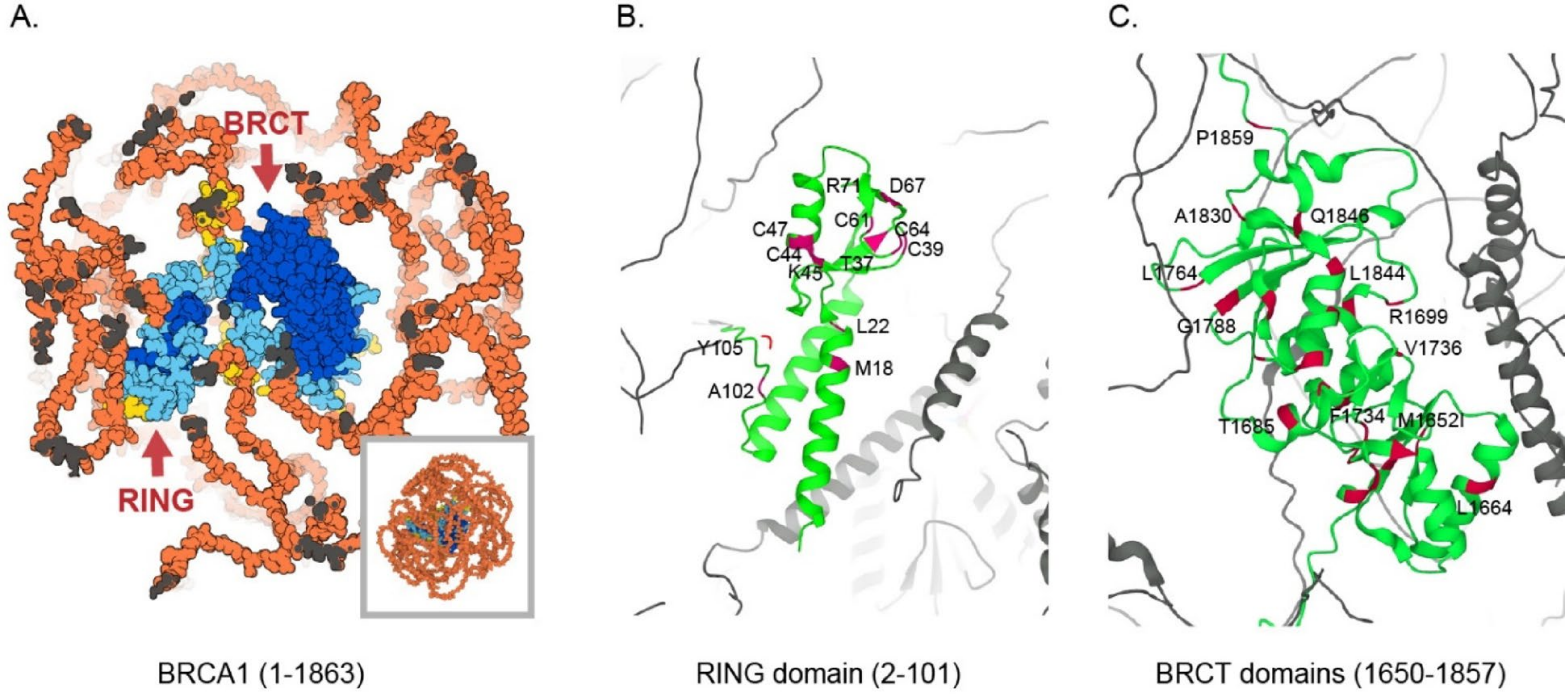
(**A**) Space filling representation of the predicted AlphaFold structure of BRCA1 (UniProt identifier P38398). The amino acids are coloured according to the AlphaFold per-residue model confidence score (pLDDT) from very high (pLDDT > 90) (dark blue), high (90 > pLDDT > 70) (light blue) to low (70 > pLDDT > 50) (yellow) and very low (pLDDT < 50) (orange). The conserved RING and BRCT domains are located in the core of the protein and embedded by presumably unstructured stretches. (**B**) Shows a ribbon of the RING domain composed of the BARD1-interacting alpha helices (lower part) and the Zn^2+^ coordinated ligase (upper part). The position of the examined variants is shown in red together with the corresponding amino acid. (**C**) Shows a ribbon of the two BRCT domains. The position of the examined variants is shown in red together with the corresponding amino acid.

We subsequently examined binding of a series of known benign, VUSs and pathogenic variants in the BRCA1 RING and BRCT domains, respectively. Figure 4 shows the predicted AlphaFold structure^28^ of the two domains within the full-length BRCA1 protein, with the positions of the included variants highlighted in the enlarged view of the domains. The RING and BRCT domains are located at the core of BRCA1, surrounded by extensive regions that are likely intrinsically disordered. Variants were selected to span the domains and to represent a range of both benign and pathogenic classifications. Moreover, they should be annotated in ClinVar or assessed in the recent BRCA1 SGE analyses^4^. The RING module includes amino acids 2–101 (ClinGen *BRCA1* Variant Curation Expert Panel) and comprises two flanking alpha helices involved in heterodimerization with BARD1 (residues 2–23 and 65–101), along with the central catalytic RING domain (residues 24–64). The BRCT domain of BRCA1 consists of two tandem BRCT repeats spanning amino acids 1650 to 1857, as defined by the ClinGen *BRCA1* Variant Curation Expert Panel. A few benign variants situated near the BRCA1 RING (Tyr105Cys) and BRCT (Pro1859Arg) domains were also included in the study.

**Fig. 5:**
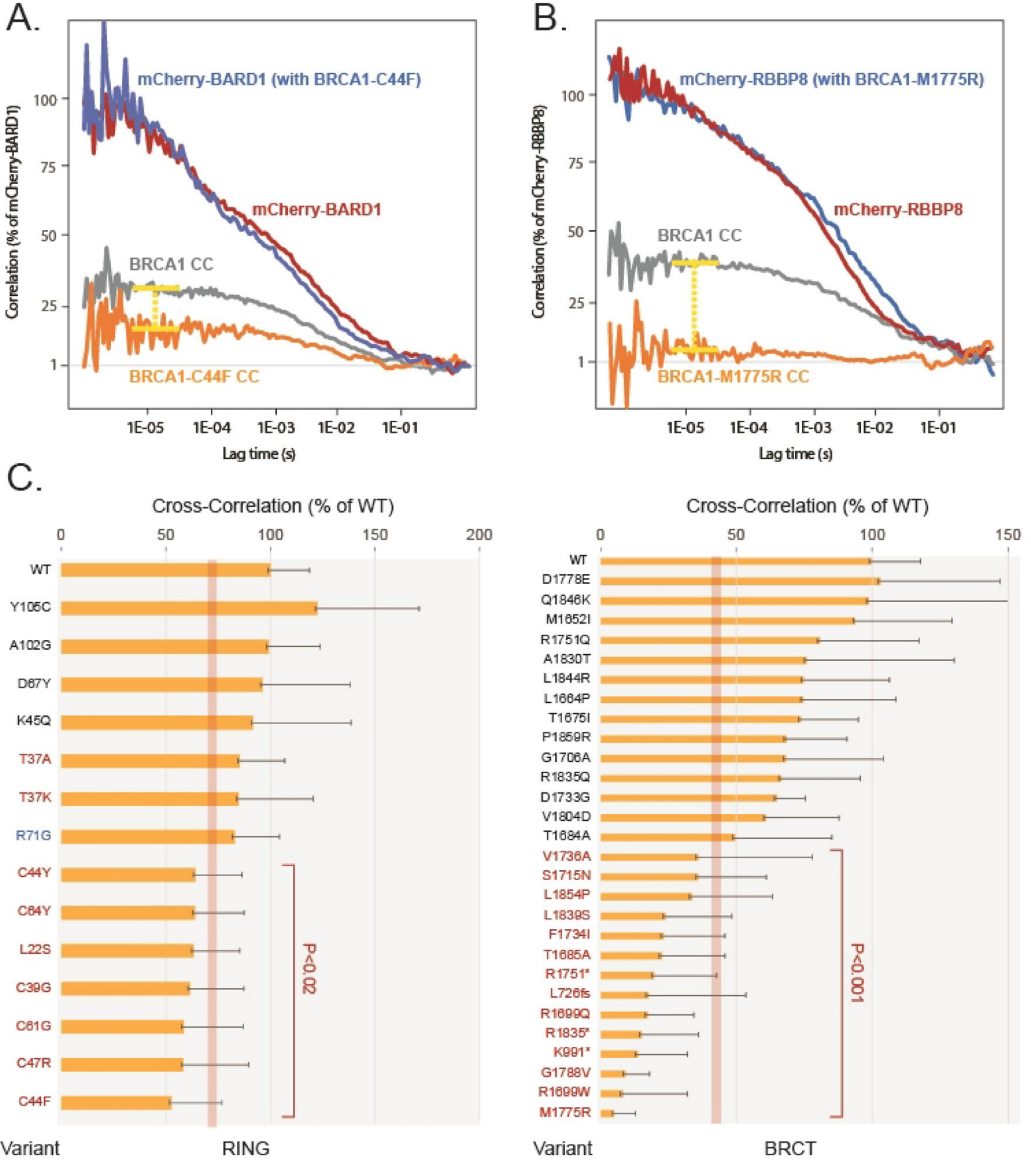
Impaired binding of *BRCA1* variants to BARD1 and RBBP8/CtIP in live cells. (**A**) Normalized mCherry-BARD1 autocorrelation and cross-correlation (CC) curves with GFP-BRCA1 wild-type or GFP-BRCA1-C44F (GFP-BRCA1 autocorrelation curves not shown). (**B**) Normalized mCherry-RBBP8 autocorrelation and cross-correlation (CC) curves with GFP-BRCA1 wild-type and GFP-BRCA1 M1775R (GFP-BRCA1 autocorrelation curves not shown). (**C**) Averaged and normalized cross-correlation values of RING variants and BRCT variants, respectively. Recordings were obtained from 5–10 different cells and in each cell, we performed at least 3 recordings of 30 s. The columns are ordered according to the reduction in binding. Benign variants or VUSs are labelled in black, whereas pathogenic variants are labelled in red. The R71G splice variant is labelled in blue. The error bars represent the STDEV and the corresponding *P* values (*t-*test, two-tailed, unequal variance) are indicated. The vertical red line in each graph shows the threshold set to define variant deleteriousness.

The graphs in Fig. 5A, B display averaged autocorrelation curves of wild-type BRCA1 along with a representative RING (Fig. 5A) or BRCT (Fig. 5B) variant, both of which exhibited reduced cross-correlation. Figure 5C shows the results from all assessed variants, expanding on the representative examples in Fig. 5A, B. The distribution and variation of plateau correlation values across individual replicates for the different variants are shown in Supplementary Fig. 2. Based on known pathogenic and benign variants, binding levels defined as less than 70% of the wild-type BRCA1 (*P* < 0.02) were used as threshold to define deleterious variants in the RING domain (Fig. 5C, red line). With the exceptions of T37A, T37K and R71G variants, pathogenic RING mutations resulted in a significant decrease in cross-correlation between GFP-BRCA1 and mCherry-BARD1 (*P* < 0.02), whereas benign variants showed no significant impact on binding (Fig. 5C; Table 1). The BRCA1-C44F variant displayed a reduction in binding to BARD1 of approximately 50%, while the remaining pathogenic variants exhibited reductions ranging from 35% to 45% relative to wild-type levels. The pathogenic R71G variant is known to affect mRNA splicing^29,30^ (Table 1) and was therefore not expected to impair BRCA1-BARD1 binding in our assay. T37A and T37K, in contrast, are located at a binding pocket near the RING ligase domain and were examined in more detail using isolated domains (see below). We also tested the M18T variant but were unable to express the protein at levels sufficient for analysis, suggesting that the mutant was unstable. This variant was also examined in the context of the isolated domain (see below).

**Table 1:** Summary of the classification of the examined BRCA1 RING and BRCT variants by FCCS, clinvar (retrieved on May 30th 2025), saturation genome editing (SGE)^4^ and AlphaMissense^2^. The location, allele frequency (retrieved from GnomAD on May 30th 2025), RsIDs and SGE scores are indicated for each variant. †M18T, T37R and H1746Q results only from lysate analyses with isolated RING and BRCT domains. LOB, loss of binding; LOF, loss of function; AMB, ambiguous; NA, not assessed.

Transcript	Protein	FCCS	ClinVar	SGE	SGE score	SGE RNA	AlphaMissense	Frequency	rs ID
RING									
c.53T > C	p.Met18Thr†	LOB	5	LOF	−1.89	−1.12	Likely pathogenic	0.000004	rs80356929
c.65T > C	p.Leu22Ser	LOB	5	LOF	−2.11	−0.79	Likely pathogenic	NA	rs80357438
c.109 A > G	p.Thr37Ala	Binding	4	Functional	0.11	0.11	Likely pathogenic	NA	rs2055253295
c.110 C > A	p.Thr37Lys	AMB	5	LOF	−2.49	−0.78	Likely pathogenic	NA	rs80356880
c.110 C > G	p.Thr37Arg†	LOB	4/5	LOF	−2.49	−0.73	Likely pathogenic	0.000001	rs80356880
c.115T > G	p.Cys39Gly	LOB	4/5	LOF	−2.22	−0.60	Likely pathogenic	NA	rs80357164
c.131G > A	p.Cys44Tyr	LOB	5	LOF	−2.40	0.05	Likely pathogenic	NA	rs80357446
c.131G > T	p.Cys44Phe	LOB	5	LOF	−2.54	−0.87	Likely pathogenic	0.000002	rs80357446
c.133 A > C	p.Lys45Gln	Binding	1	Functional	−0.27	−0.14	Ambiguous	0.000024	rs769650474
c.139T > C	p.Cys47Arg	LOB	5	LOF	−1.84	−0.50	Likely pathogenic	NA	rs80357370
c.181T > G	p.Cys61Gly	LOB	5	LOF	−1.74	−0.27	Likely pathogenic	0.000019	rs28897672
c.191G > A	p.Cys64Tyr	LOB	5	LOF	−2.05	0.12	Likely pathogenic	0.000001	rs55851803
c.199G > T	p.Asp67Tyr	AMB	1	Functional	−0.08	0.04	Likely benign	0.000083	rs80357102
c.211 A > G	p.Arg71Gly	AMB	5	LOF	−1.49	−2.79	Likely pathogenic	0.000001	rs80357382
c.305 C > G	p.Ala102Gly	Binding	1	NA	NA	NA	Likely benign	0.000002	rs80357190
c.314 A > G	p.Tyr105Cys	Binding	1	NA	NA	NA	Likely benign	0.000181	rs28897673
BRCT									
c.2176_2177del	Leu726fs	LOB	5	NA	NA	NA	NA	NA	rs397508945
c.2971 A > T	p.Lys991*	LOB	5	NA	NA	NA	NA	NA	rs886040090
c.4956G > A	p.Met1652Ile	Binding	1	Functional	0.14	−0.44	Ambiguous	0.014620	rs1799967
c.4991T > C	p.Leu1664Pro	Binding	1	Functional	−0.01	−0.22	Likely benign	0.000004	rs80357314
c.5024 C > T	p.Thr1675Ile	Binding	1	Functional	0.04	−0.03	Likely benign	0.000012	rs150729791
c.5050 A > G	p.Thr1684Ala	Binding	2	Functional	−0.65	0.15	Ambiguous	NA	rs879255491
c.5053 A > G	p.Thr1685Ala	LOB	5	LOF	−1.84	−0.30	Ambiguous	NA	rs80356890
c.5095 C > T	p.Arg1699Trp	LOB	5	NA	NA	NA	Likely pathogenic	0.000008	rs55770810
c.5096G > A	p.Arg1699Gln	LOB	5	NA	NA	NA	Likely pathogenic	0.000019	rs41293459
c.5117G > C	p.Gly1706Ala	Binding	1	Functional	−0.05	−0.20	Ambiguous	0.000046	rs80356860
c.5144G > A	p.Ser1715Asn	LOB	5	LOF	−2.29	−0.51	Likely pathogenic	0.000001	rs45444999
c.5198 A > G	p.Asp1733Gly	Binding	1	Functional	0.17	0.19	Likely benign	0.000056	rs80357270
c.5200T > A	p.Phe1734Ile	LOB	3/4/5	LOF	−2.26	−0.65	Likely pathogenic	NA	rs80356957
c.5207T > C	p.Val1736Ala	LOB	5	LOF	−1.60	0.16	Likely pathogenic	0.000008	rs45553935
c.5238 C > G	p.His1746Gln†	LOB	3	Intermediate	−0.90	0.05	Likely pathogenic	NA	rs786202389
c.5251 C > T	p.Arg1751*	LOB	5	LOF	−1.88	−1.27	NA	0.000004	rs80357123
c.5252G > A	p.Arg1751Gln	Binding	1	Functional	−0.15	−0.18	Likely benign	0.000048	rs80357442
c.5324T > G	p.Met1775Arg	LOB	5	LOF	−1.39	−0.64	Likely pathogenic	0.000003	rs41293463
c.5334T > A	p.Asp1778Glu	Binding	3	Functional	−0.21	−0.24	Likely benign	NA	rs754152768
c.5363G > T	p.Gly1788Val	LOB	5	LOF	−1.68	−0.39	Likely pathogenic	0.000001	rs80357069
c.5411T > A	p.Val1804Asp	Binding	1	Functional	0.43	−0.04	Likely benign	NA	rs80356920
c.5488G > A	p.Ala1830Thr	Binding	2/3	Functional	0.17	−0.23	Likely benign	0.000001	rs80357393
c.5503 C > T	p.Arg1835*	LOB	5	LOF	−2.30	−1.41	NA	0.000012	rs41293465
c.5504G > A	p.Arg1835Gln	Binding	2/3	Intermediate	−0.91	0.70	Ambiguous	0.000014	rs273902776
c.5516T > C	p.Leu1839Ser	LOB	5	LOF	−2.49	−0.70	Likely pathogenic	NA	rs398122702
c.5531T > G	p.Leu1844Arg	Binding	1	Functional	−0.06	−1.12	Likely benign	0.000009	rs80357323
c.5536 C > A	p.Gln1846Lys	Binding	1/3	Functional	−0.09	−0.12	Likely benign	0.000001	rs80356873
c.5561T > C	p.Leu1854Pro	LOB	3/4	LOF	−1.34	−0.92	Likely pathogenic	NA	rs80356996
c.5576 C > G	p.Pro1859Arg	Binding	1	NA	NA	NA	Likely benign	0.000061	rs80357322

Pathogenic variants in the BRCT module clearly impacted RBBP8 binding, and the binding data were consistent with ClinVar classification, SGE scores and AlphaMissense predictions (Fig. 5C; Table 1). Pathogenic variants such as M1775R, G1788V, and R1699W strongly reduced RBBP8 binding and were nearly indistinguishable from the truncating variants L726fs and K991*. Several benign BRCT variants also showed reduced binding, and the extent of binding reduction appears to vary along a continuous spectrum, as illustrated in Fig. 5C. Therefore, to distinguish deleterious BRCT variants, binding defined as less than 40% of the wild-type level was used as threshold (*P* < 0.001) (Fig. 5C, red line). Table 1 summarizes the results from FCCS analyses and shows the corresponding ClinVar classification, SGE scores and AlphaMissense predictions. The correlation to previous massively parallel screening data^4,19^ is shown in Supplementary Fig. 3, while Supplementary Table 1 provides a comprehensive comparison of the results of our study with previously reported assessments of the same BRCA1 variants^4,19,31–37^, including all studies approved by the ClinGen *BRCA1* Variant Curation Expert Panel^38,39^ at the time of this publication.

### FCCS analyses of isolated BRCA1 RING and BRCT domain variants in cell lysates

**Fig. 6:**
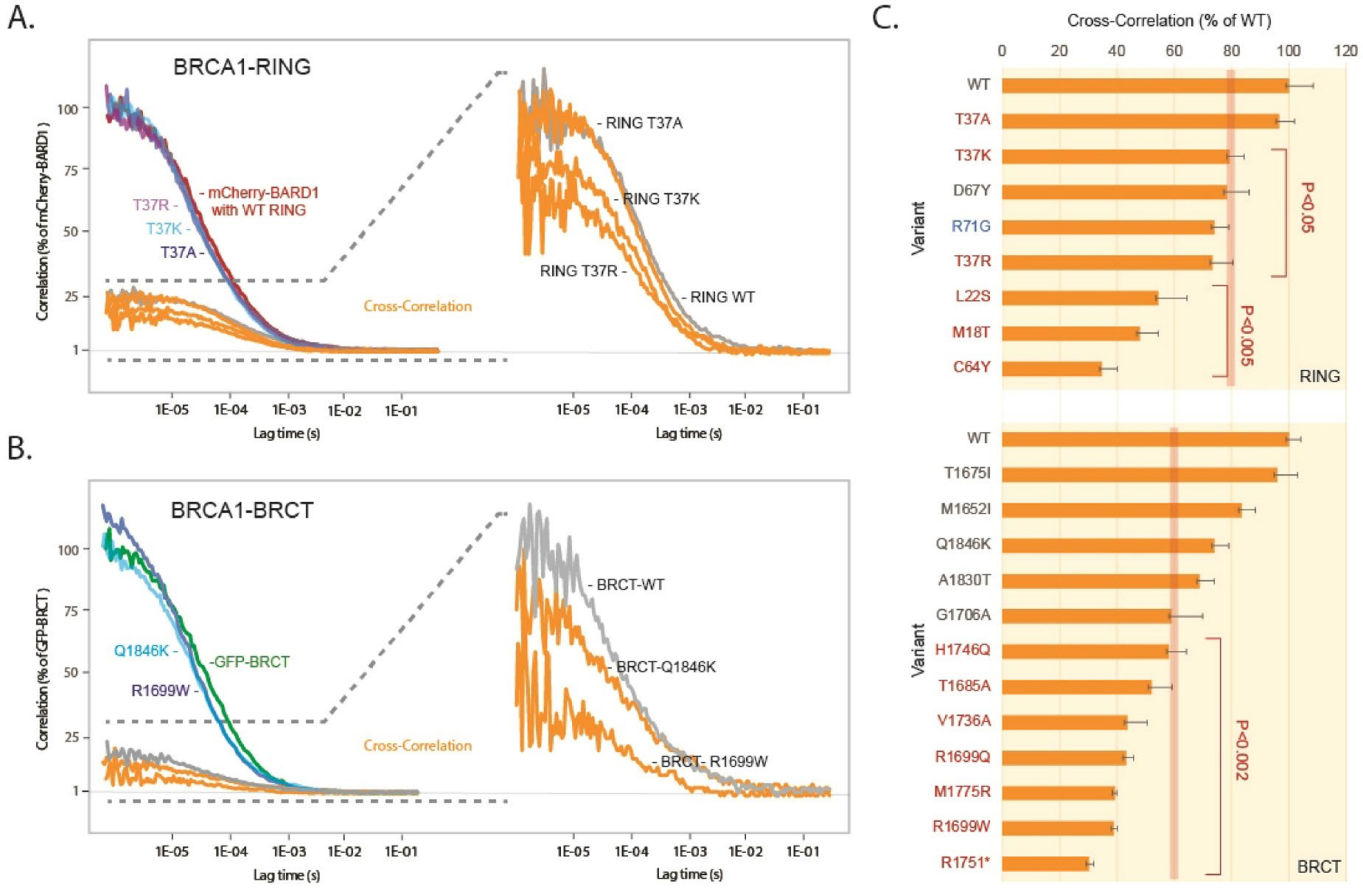
BRCA1 variant analysis employing isolated RING and BRCT domains in cell lysates. (**A**) Cells were transfected for 24 h with either GFP-RING, GFP-RING-T37A, GFP-RING-T37K or GFP-RING-T37R in combination with mCherry-BARD1 before they were solubilized, and the lysates were examined using FCCS. The panel shows the normalized mCherry-BARD1 autocorrelation curve (red) and corresponding cross-correlation curve with wild-type RING (grey), as well as the mCherry-BARD1 autocorrelation and cross-correlation curves (orange) from the analyses of the GFP-tagged RING-T37A (dark blue), T37K (light blue) or T37R (purple) variants. The right panel shows an enlarged view of the cross-correlation curves for each variant. (**B**) Normalized autocorrelation curve of GFP-BRCT (green) and the corresponding cross-correlation curve (grey) with RBBP8, as well as the GFP-BRCT autocorrelation and cross-correlation curves (orange) from the analyses of the GFP-tagged BRCT-Q1846K (light blue) and R1699W (dark blue) variants. The right panel shows an enlarged view of the cross-correlation curves for each variant. (**C**) Normalized averaged cross-correlation values from RING and BRCT variants. The columns are ordered according to the reduction in binding. Benign variants or VUSs are labelled in black, whereas pathogenic variants are labelled in red. The R71G splice variant is labelled in blue. The results are derived from three independent experiment. The error bars indicate the STDEV. The error bars represent the STDEV and the corresponding *P* values (*t-*test, two-tailed, unequal variance) are indicated. The intra-assay variations are shown in Supplementary Fig. 4.

We recently developed an FCCS protocol employing cell lysates to complement live cell readings^27^. While lysates lack the spatial resolution of live cells, their reduced spatial constraints and ability to be diluted enable more precise detection of subtle changes in binding. Moreover, unlike single-cell live analysis, this approach measures averaged signals from a population of cells, resulting in lower variability across replicates. Briefly, cells were transfected with isolated BRCA1 GFP-RING or GFP-BRCT domains in combination with mCherry-BARD1 or mCherry-RBBP8, respectively, followed by iso-osmolar lysis using a buffer containing non-ionic detergent to release the cytoplasmic content. Measurements were performed on a drop of lysate placed at the bottom of the same type of glass dishes used for live cell recordings. For each variant, we performed 12 measurements in 3 biological replicates. Correlation values ranged from 1.5 to 2.2, approximately 50-fold higher than those observed in live cells. For the wild-type RING domain, cross-correlation was 25% of the mCherry-BARD1 autocorrelation, while for the BRCT domain, the cross-correlation with mCherry-RBBP8 was 17% (Fig. 6A, B and Supplementary Fig. 4 A). The averaged results from the analysis of selected variants are shown in Fig. 6C and the corresponding replicate data are presented in Supplementary Fig. 4B, C. Based on known pathogenic and benign variants, binding levels defined as less than 80% of the wild-type BRCA1 (*P* < 0.05) were used as threshold to define deleterious variants in the RING domain. The isolated domain largely recapitulated the results obtained with full-length BRCA1, and as illustrated in Fig. 6A, the higher accuracy of the assay enabled detection of subtle differences in the binding of T37K, T37R and T37A variants. We detected a minor (20%) but significant reduction in binding for the pathogenic T37K variant (*P* < 0.05), while the T37R variant showed a 30% decrease in binding (*P* < 0.05). Based on the combined results from live-cell and lysate-based FCCS experiments, the T37K variant is classified as ambiguous, exhibiting robust binding to BARD1 in live cells but reduced binding in lysates. Additionally, the D67Y variant showed a 22% reduction in binding (*P* < 0.05). Moreover, unlike live cells, the M18T variant in the isolated domain was adequately expressed and found to exhibit reduced binding, classifying it as deleterious.

For the BRCT domain, we also found that pathogenic variants showed results consistent with the live cell analysis (Fig. 6B, C). Compared to the RING domain, BRCT variants generally had slightly more pronounced effects on binding. Consistent with observations in live cells, even benign variants showed a reduction in binding to RBBP8. Therefore, to distinguish deleterious BRCT variants, binding defined as less than 60% of the wild-type level was used as threshold (*P* < 0.002).

Taken together, our results suggest that isolated structural modules provide a simple and effective way to classify genetic variants.

### Application of FCCS analyses to MSH2-MSH6 and Menin-JUND heterodimers

**Fig. 7:**
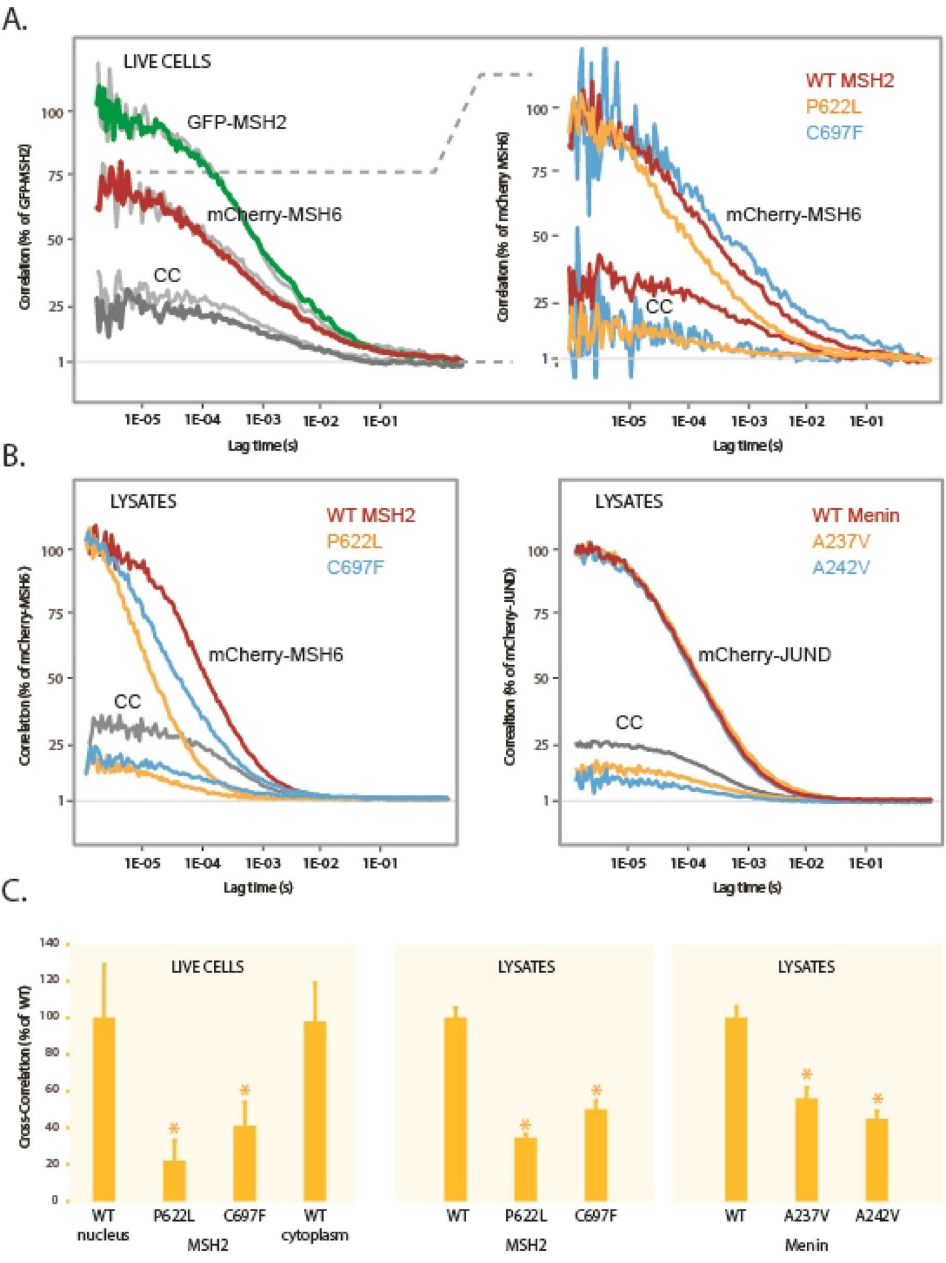
FCCS analyses of MSH2 and Menin. (**A**) Nuclear autocorrelation (green and red) and cross-correlation (CC) curves (dark grey) from cells transfected with GFP-MSH2 and mCherry-MSH6. The light grey curves show the same analyses where the focal volume was positioned in the cytoplasm. The enlarged section on the right displays analyses of the pathogenic *MSH2* variants; P622L and C697F. (**B**) FCCS analyses of the same MSH2 constructs were performed in cellular lysates (left panel) and with GFP-Menin and mCherry-JUND (right panel), or with JUND in combination with Menin variants A237V or A242V. (**C**) Normalized cross-correlation results from three independent experiments. Error bars represent the STDEV and asterisks indicate *P* < 0.05 (*t*-test, two-tailed, unequal variance).

To explore whether FCCS could be applied to other structural domains, we performed assays using MSH2 and MSH6, proteins involved in hereditary non-polyposis colorectal cancer (HNPCC), as well as Menin, which is encoded by the multiple endocrine neoplasia (*MEN1*) gene, in combination with JUND. The analyses were performed as described for BRCA1, and in live cells we observed a strong cross-correlation of 34% between MSH2 and MSH6 in both the nucleus and cytoplasm (Fig. 7A). Cross-correlation was significantly reduced by the insertion of pathogenic *MSH2* variants; P622L and C697F^9,40^ (Fig. 7A and C, left panel). In lysates, cross-correlation was slightly lower (27%), but as observed in live cells, the two pathogenic variants caused a significant reduction in cross-correlation (Fig. 7B, left panel, and 7 C, middle panel). In lysates, we also observed a shift to the left in the correlation curves, suggesting that the variants are likely excluded from a larger protein assembly. The interaction between Menin and JUND was assessed in lysates, revealing a cross-correlation of 21% (Fig. 7B, right panel). Two Menin variants - A237V and A242V^41^, categorised in ClinVar as VUS and likely pathogenic, respectively, and both classified as likely pathogenic by AlphaMissense, reduced binding to approximately 50% of wild-type levels (Fig. 7C, right panel).

Taken together, we infer that FCCS is applicable for variant classification in various structural domains.

## Discussion

Key cellular processes, including transcription, mRNA processing and transport, protein synthesis, signalling events, and metabolism, depend on the orchestration of protein assemblies with varying degrees of complexity. While some protein complexes may consist of homomeric structures, they more often involve an interplay of distinct proteins. Binding is typically governed by conserved domains with well-defined structures, though it can also be mediated by intrinsically disordered regions, albeit less commonly^42,43^. Many monogenic diseases are associated with variants that disrupt the formation of specific functional assemblies^44–46^, e.g. hereditary cancer syndromes, developmental abnormalities resulting from disruptions in cytoskeletal or ciliary architecture, as well as metabolic, mitochondrial diseases, and late-onset conditions like Amyotrophic Lateral Sclerosis^47^. Recent data suggest that pathogenic mutations often cluster at protein interfaces, highlighting the potential utility of analysing protein interactions to better characterise the pathogenic significance of missense variants^42^. Generally, missense variants affect protein structure by inducing misfolding, which can sometimes lead to the degradation of the affected proteins. Based on the idea that analysing complex formation could serve as a sensitive and broadly applicable approach for identifying pathogenic variants, we used Fluorescence Correlation and Cross-Correlation Spectroscopy to rapidly and precisely assess defects in in vivo protein complex formation.

The main limitations of FCS and FCCS stem from the need to tag proteins with fluorophores, which can sometimes affect their folding and biological activity. BRCA1 can be tagged without significant loss of biological activity, and GFP-BRCA1 has previously been used to characterise its role at double-strand break (DSB) sites^48^. Consistent with earlier studies, our initial characterisation of the GFP-BRCA1 construct showed that it localised to characteristic nuclear foci and formed functional complexes with other proteins. The association between BRCA1 and BARD1, as well as RBBP8/CtIP, was close to 30%, comparable to that observed with a mCherry-GFP fusion protein, indicating that the binding is consistent and stable (Supplementary Fig. 1). Due to photobleaching, misfolding or fluorescent proteins existing in “off” or in dark states^49^, cross-correlation is not expected to reach 100%. In our experience, it is helpful to separate the protein of interest from the fluorophore introducing a short, flexible linker sequence^27^. Furthermore, we have found that the relative abundance of the proteins should be as similar as possible, and it is important to ensure that measurements are taken within the linear range of the photodetector. Transient expression systems can produce relatively high concentrations of expressed proteins; therefore, cells with lower expression levels should generally be selected for analysis. Whenever possible and appropriate, we prefer lysate-based analysis, as the protein concentration can be precisely controlled, thereby ensuring optimal correlation amplitude and assay accuracy. Moreover, lysates provide a bulk analysis, which helps reduce the pronounced cell-to-cell variation we observed for some variants in single-cell live analysis. Although these approaches require specialised microscopy equipment and technical expertise, data acquisition is rapid, with individual measurements completed within minutes, making the assay well suited to both research and clinical applications due to its speed and reproducibility. Finally, it is important to emphasise that this assay should only be considered for its positive predictive value (reduced binding), since protein binding can still occur normally despite issues such as loss of enzymatic activity or improper splicing, as described below.

In general, the FCCS findings were consistent with the current SGE^4,19^ and screening by E3 ligase activity in combination with two-hybrid testing^19^, ClinVar, and AlphaMissense classifications of the examined variants. Reduced binding was observed with pathogenic or ambiguous variants, both in live cells and in isolated domains in lysates. Two known pathogenic variants, T37K and R71G, exhibited normal binding in live cells. T37 is a solvent-exposed residue positioned within a small cavity between the BRCA1-BARD1 heterodimerization interface and the catalytic site^50^. T37K showed a small but significant effect on BRCA1-BARD1 binding in lysates, yet displayed normal binding in live cells, highlighting the limitations of live cell analysis and our preference for using lysate-based assays. On the other hand, the T37R variant exhibited a greater loss of binding in lysates, likely due to arginine’s larger number of electrostatic interactions perturbing the overall structure to a greater extent. Of note, in agreement with the SGE score, T37A was defined as neutral in our assay, however the variant was recently classified as likely pathogenic in ClinVar, confirming that protein loss of function can occur in the presence of normal binding. As previously mentioned, R71G has been documented to reduce splicing^29^ and the resulting decrease in mRNA expression is also reflected in the SGE analysis (Table 1). Since the constructs we used are intron-less, we expected them to behave similarly to the wild-type, although we did observe a small reduction in binding. Finally, D67Y also exhibited a small reduction in binding in lysates. Although it is generally classified as benign, some reports have suggested that D67Y may be hypomorphic^51–53^, prompting us to label the variant as ambiguous.

The domain-specific analyses reconciled the results from live cells but also revealed that, overall, the variants had a slightly greater impact on binding in the isolated domains. This was particularly evident for variants in the conserved BRCT domains, where even benign variants reduced binding to RBBP8. In fact, sorting the variants by their binding shows that they form a continuum from wild-type activity to severe reduction in binding. Pathogenic variants were clearly more severe than benign ones and could be identified by a combination of reduced binding and statistical significance. The lower impact on binding in live cell analyses underscores the biological significance of larger molecular assemblies, in the sense that the concerted action of many factors may stabilise binding of each individual factor. Moreover, in the specific case of BRCA1 the results could point to a role for the long stretches of intrinsically disordered sequences. The results also indicate that deleterious variants in the future may be better qualified by quantitative measures rather than a binary (likely)benign – (likely)pathogenic way. An example is the T1684A variant that, despite being classified as neutral according to our FCCS data and the SGE score, showed reduction in binding of approximately 50% and therefore could be envisioned to be harmful for susceptible persons. Recent data indicate that protein-folding chaperones may overcome the pathogenicity of variants in the BRCA1 C-terminal (BRCT) domain. HSP70 binds pathogenic BRCA1-BRCT variants, and the magnitude of binding has been correlated to loss of folding and function^54^. If the chaperone activity varies in the population, some individuals may be particularly susceptible to variants that are otherwise classified as benign. Chemical chaperones have moreover successfully been applied to re-establish the function of mutant proteins in monogenic diseases^54–57^ and in this scenario quantitative methods such as FCCS may be helpful to identify relevant variants and monitor the effect of the chaperone.

Finally, we examined the feasibility of employing FCCS to analyse other domains, including variants in *MSH2* and Menin, respectively. Since both proteins are significantly smaller than BRCA1, live cell analyses were considerably easier to perform due to a much higher transfection efficiency. MSH2 is composed of five structural domains, including a DNA mismatch binding domain, a connector domain, a lever domain with an incorporated Clamp domain, and a C-terminal ATPase domain^58^. Notably, the pathogenic P622L and C697F variants in *MSH2* are both located within the ATPase domain, positioned on two distinct beta strands. MSH2 and MSH6 exhibited a high cross-correlation (34%), however binding was clearly reduced by the two pathogenic variants. Interestingly, we also observed that impaired heterodimerization led to a significant increase in the diffusion constant of cytoplasmic MSH2, suggesting the involvement of additional factors.

On the other hand, Menin is a single domain scaffold protein that regulates gene transcription and cell signalling^59^. Interestingly, Menin adopts a shape reminiscent of a curved hand, with JUND binding to a central pocket that spans residues 27–47, which is relatively distant from the mutated residues R237 and R242. Although JUND binds at residues 27–47, mutations at R237 and R242 still impact Menin-JUND binding, underscoring that the entire amino acid composition of this type of protein may have been fine-tuned during evolution and supporting the concept of structure-based analysis for classifying genetic variants. These two examples further illustrate the broader potential of FCCS to investigate the impact of variants in proteins beyond BRCA1. This promising approach lays the groundwork for future studies, although establishing robust thresholds for clinical interpretation will benefit from additional data from known benign and pathogenic variants.

Taken together, FCCS is reproducible and fast, requiring only a confocal microscope and laboratory space for cell transfection. Including the time for transfection and recordings, the entire analysis can be completed in just two workdays, making FCCS an appealing option for the clinical environment. We do not envision FCCS as a tool for systematic functional screens like those conducted for BRCA1^4^ or MSH2^60^ variants, however, the technology holds promise for the functional assessment of individual patient variants across a wide range of monogenic diseases, particularly where no other functional test is available. The specific cut-off level obviously needs to be established between known pathogenic and benign variants during clinical implementation for other monogenic diseases. In summary, we examined four different protein interactions involving various structured domains and successfully identified pathogenic variants in each case. Thus, FCCS may serve as a potential generic approach for functional variant assessment, providing additional information to supplement the current ACMG/AMP variant classification efforts.

## Supplementary Information

Below is the link to the electronic supplementary material.

Supplementary Material 1

Supplementary Material 2

## Data Availability

The datasets generated during the current study are available from the corresponding author on reasonable request.
