# Supplementary material for: Functional assessment of protein variants in structured domains by fluorescence cross-correlation spectroscopy
Source: Sci Rep. 2026 Jan 2;16:4554. doi: 10.1038/s41598-025-34563-6 (PMC12867984; doi:10.1038/s41598-025-34563-6)
Supplement: Supplementary file 1 — Supplementary Material 1 [file 41598_2025_34563_MOESM1_ESM.docx]

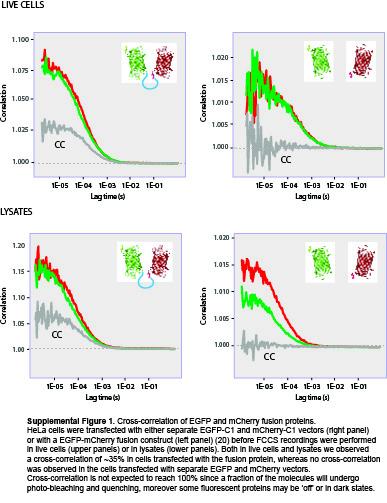


**Supplementary Fig. 1.** Cross-correlation analysis of GFP and mCherry fusion proteins. HeLa cells were transfected with either a mCherry-GFP fusion construct (left panels) or separate GFP-C1 and mCherry-C1 plasmids (right panels). FCCS measurements were conducted in both live cells (upper panels) and cell lysates (lower panels). In both conditions, cells expressing the fusion protein showed a cross-correlation (CC) of approximately 33%, while no cross-correlation was detected in cells transfected with the separate GFP and mCherry vectors. A 100% cross-correlation is not expected, as some molecules are subject to photobleaching, quenching, or may exist in non-fluorescent (dark) states.

**
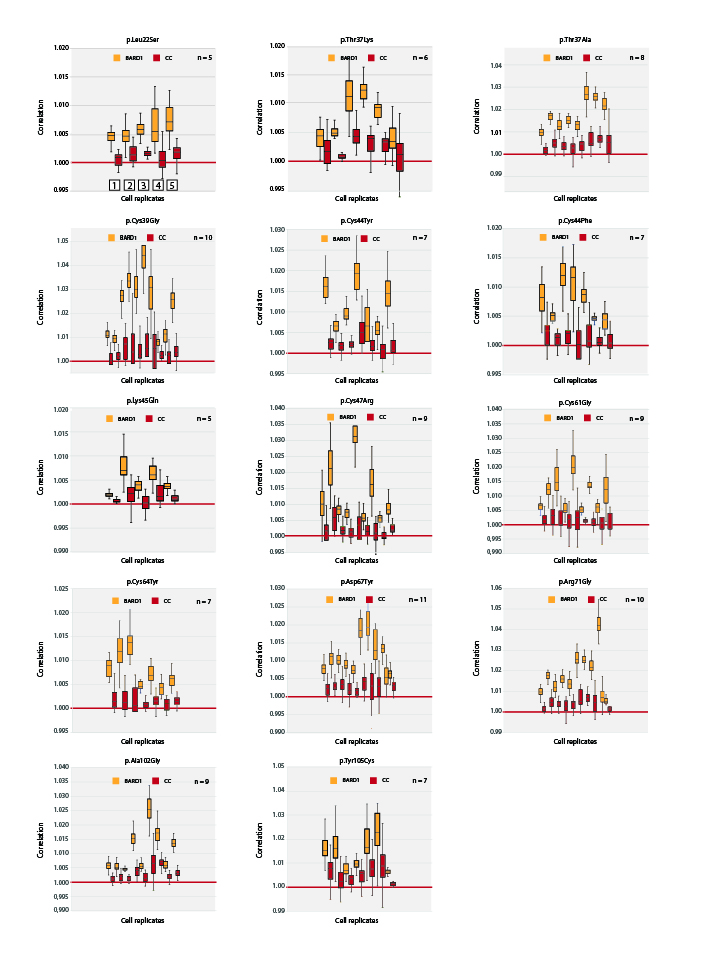
**

**
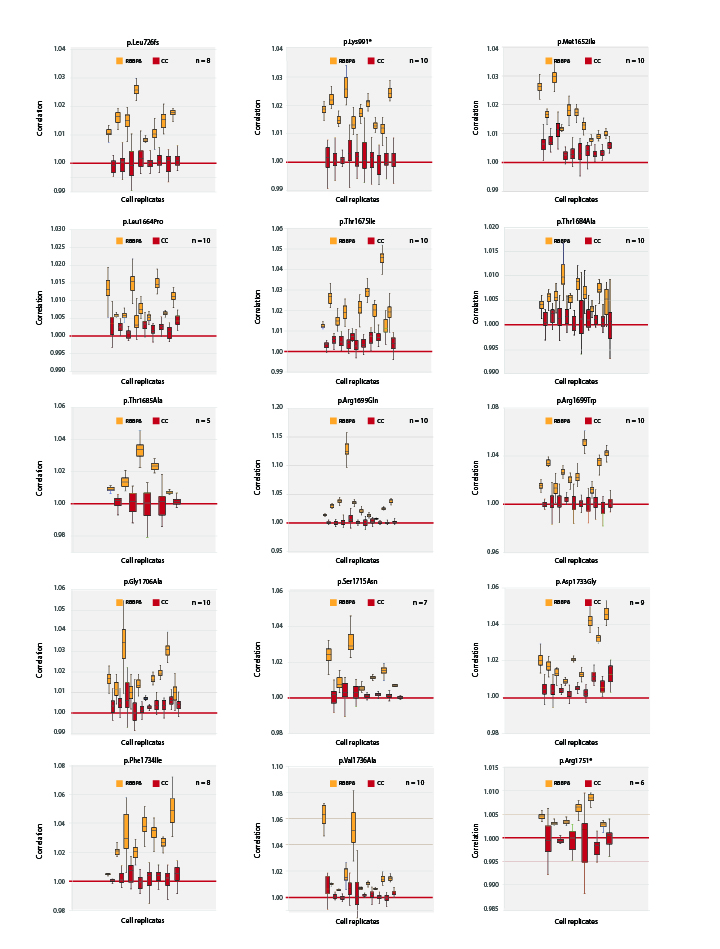
**


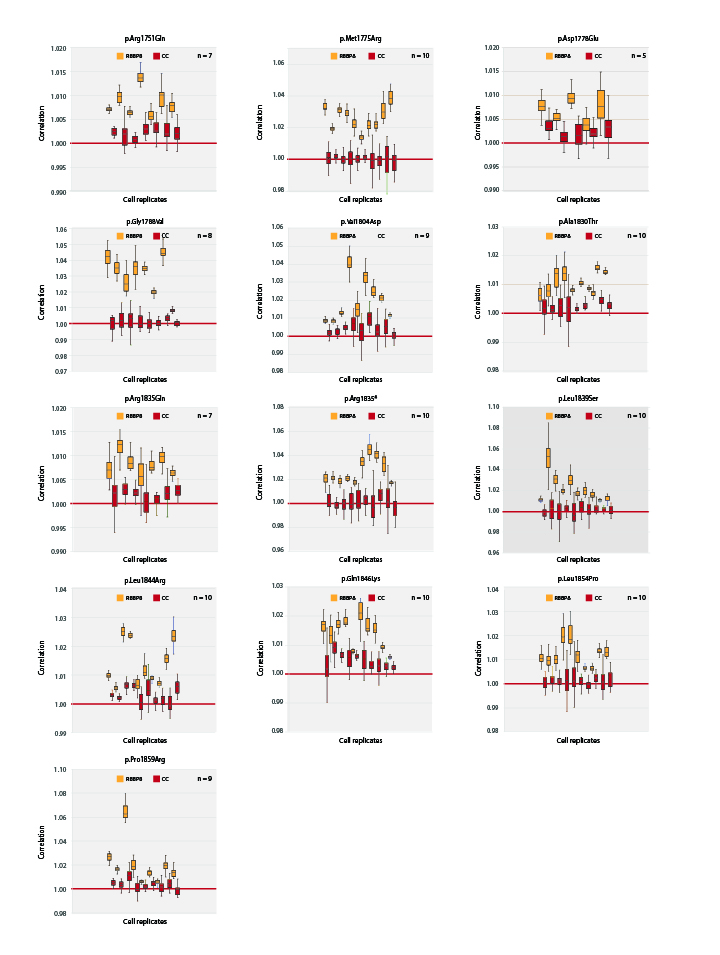
**Supplementary Fig. 2.** Summary of autocorrelation and cross-correlation data from the *BRCA1* variant analysis. For each tested variant, the charts display the distribution and variability of 20 correlation values obtained across time points ranging from 8.00E-06 – 4.00E-04. Autocorrelation values for BARD1 and RBBP8 are indicated in orange, while corresponding cross-correlation (CC) values are shown in red. Individual measurements, based on 5–10 replicates (cells), are presented side by side, as illustrated in the first chart.


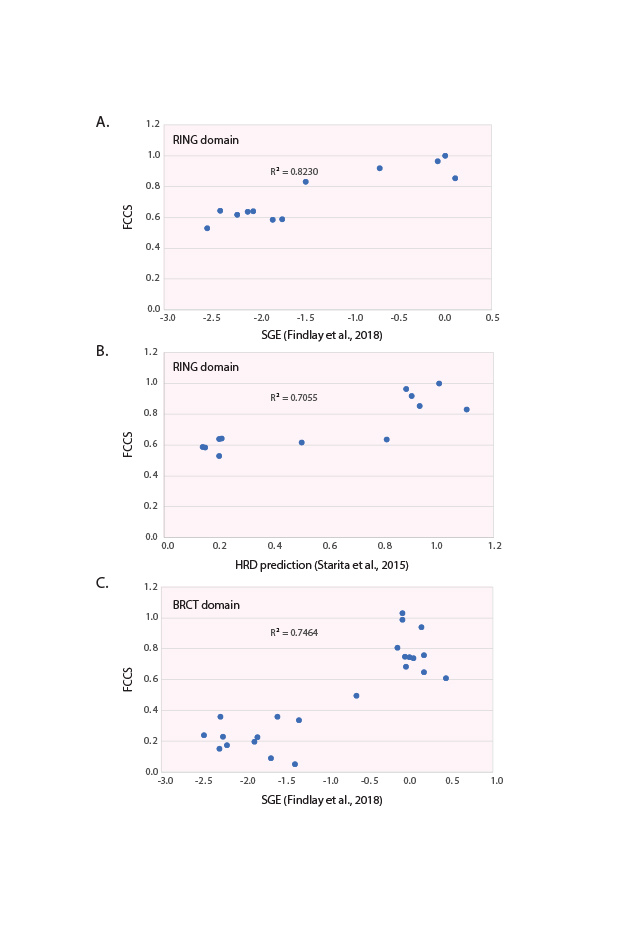


**Supplementary Fig. 3.** (**A**) Correlation between variant effect derived from FCCS data and saturation genomic editing (SGE) data (Findlay et al., 2018) for variants in the RING domain. (**B**) Correlation between variant effect derived from FCCS data and HRD activity data (Starita et al., 2015) for variants in the RING domain. (**C**) Correlation between variant effect derived from FCCS data and SGE data (Findlay et al., 2018) for variants in the BRCT domain. R^2^ values represent the Pearson correlation coefficient.


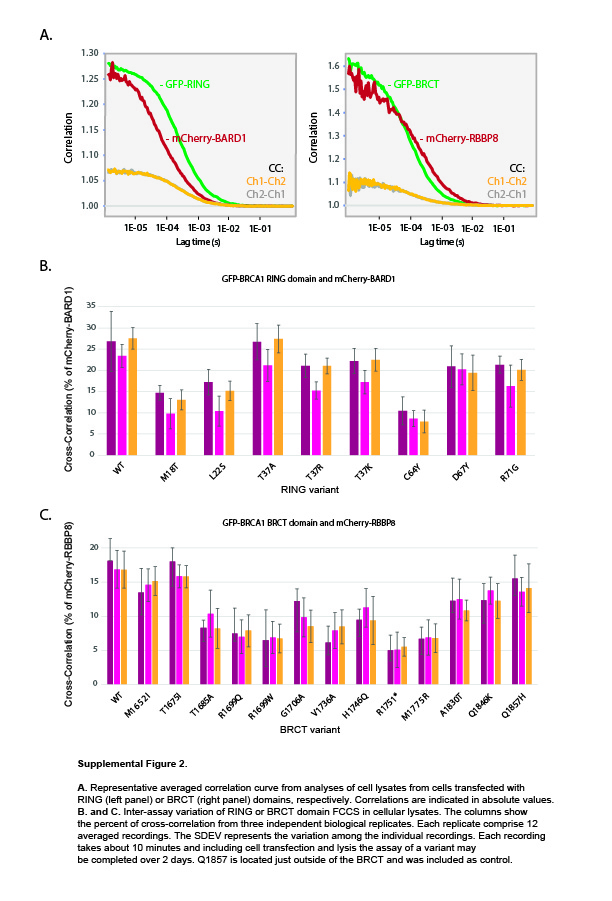


**Supplementary Fig. 4.** (**A**) Representative averaged correlation curves from analyses of lysates from cells transfected with RING (left panel) or BRCT (right panel) domains, respectively. Correlation values are shown as absolute values. (**B**) and (**C**) Inter-assay variation of FCCS measurements for the RING and BRCT domains in cellular lysates. Bars represent the percentage of cross-correlation from three independent biological replicates, each comprising 12 averaged recordings. STDEV indicates the variation among individual recordings. Each recording takes approximately 10 minutes, and including cell transfection and lysis, the assay for a given variant can be completed within two days. Q1857, located just outside the BRCT domain, was included as a control.

**Supplementary Table 1.** Comparison of functional categorizations of the examined BRCA1 RING and BRCT variants by FCCS, ClinVar (retrieved on May 30^th^ 2025) AlphaMissense and the nine most relevant functional studies with a significant overlap of assessed variants. Variants are categorized by functional studies as neutral (green), deleterious (red), intermediate (yellow) or ambiguous (grey). The studies highlighted in blue have been approved by ClinGen *BRCA1* Variant Curation Expert Panel by the time of this publication. NA, not assessed.
